# Correction: *TP53* drives invasion through expression of its Δ133p53β variant

**DOI:** 10.7554/eLife.107369

**Published:** 2025-05-02

**Authors:** Gilles Gadea, Nikola Arsic, Kenneth Fernandes, Alexandra Diot, Sebastien Joruiz, Samer Abdallah, Valerie Meuray, Stéphanie Vinot, Christelle Anguille, Judit Remenyi, Marie P Khoury, Philip R Quinlan, Colin A Purdie, Lee B Jordan, Frances V Fuller-Pace, Marion de Toledo, Maïlys Cren, Alastair M Thompson, Jean-Christophe Bourdon, Pierre Roux

**Keywords:** Human

Gadea G, Arsic N, Fernandes K, Diot A, Joruiz SM, Abdallah S, Meuray V, Vinot S, Anguille C, Remenyi J, Khoury MP, Quinlan PR, Purdie CA, Jordan LB, Fuller-Pace FV, de Toledo M, Cren M, Thompson AM, Bourdon J-C, Roux P. 2016. TP53 drives invasion through expression of its Δ133p53β variant. *eLife* **5**:e14734. doi: 10.7554/eLife.14734.

Published 15 September 2016

We were notified by eLife journal of three errors on Figure 4 panel B

1. The first one is the HCT116 image which is the duplication of the HCT116 image published in Figure 4A PLOS One 2012; 7 (11): e48344 and indicated as HCT116. This error has been corrected by replacing the originally published HCT116 image by a new HCT 116 image.

2. The second one is the SW480 image which is the duplication of the SW480 image published in Figure 4A PLOS One 2012; 7 (11): e48344. In this article, it was established that treatment (PDGF +Y27632) did not affect E-Cadherin labelling, meaning that this labelling is identical between SW480 cells cultured or not with PDGF +Y27632, as shown in Figure 4A. The image published in Figure 4B eLife therefore effectively shows E-Cadherin labelling on the cell surface in SW480. This error has been corrected by replacing the originally published SW480 image by a new SW480 image.

3. The third one is the LoVo image which is the duplication of the SW620 in figure 4A PLOS One 2012; 7 (11): e48344. This is clearly an inversion between the SW620 and LoVo images in Figure 4B eLife. This image shows the absence of E-Cadherin labelling on the surface of SW620 round cells, the same absence of labelling being observed on LoVo round cells. The images of unlabelled round LoVo and SW620 cells look very similar, which may explain the inversion of the images during the processing step. This error has been corrected by replacing the originally published LoVo image by a new SW620 image.

We predict that these duplications occurred while selecting images from our collection. It was an unintentional error to include images that are identical to those previously published in PLOS One 2012; 7 (11): e48344. However, these mistakes do not affect either the results or interpretation of this experiment, nor the conclusions of the original article.

The corrected Figure 4 is shown here:

**Figure 1:**
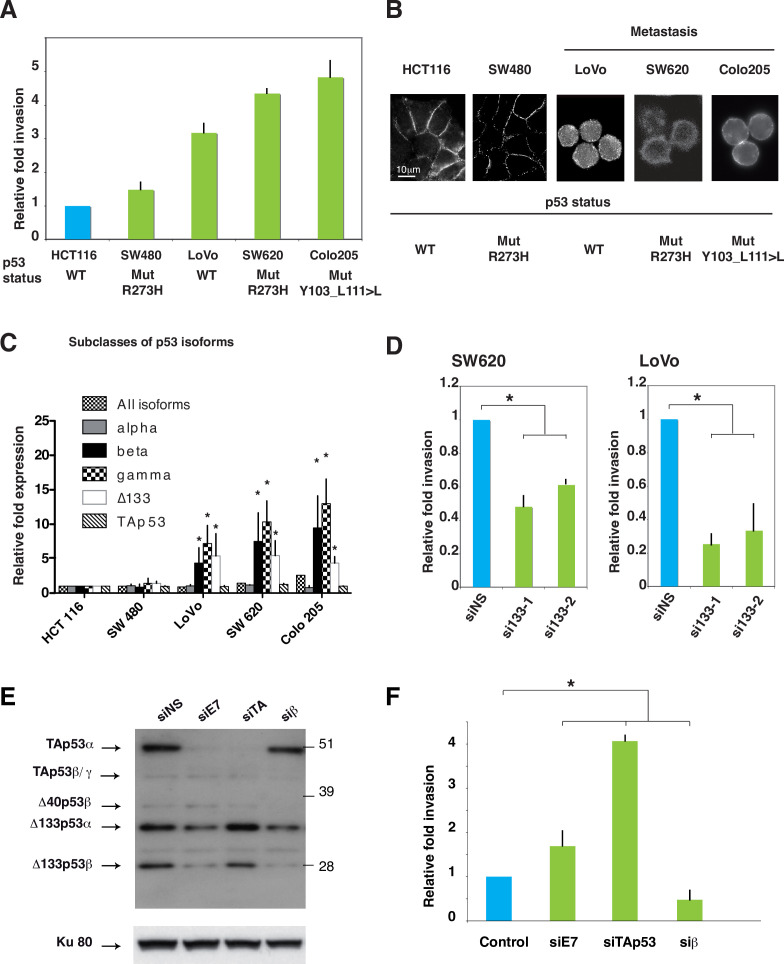


The originally published Figure 4 is shown for reference:

**Figure 2:**
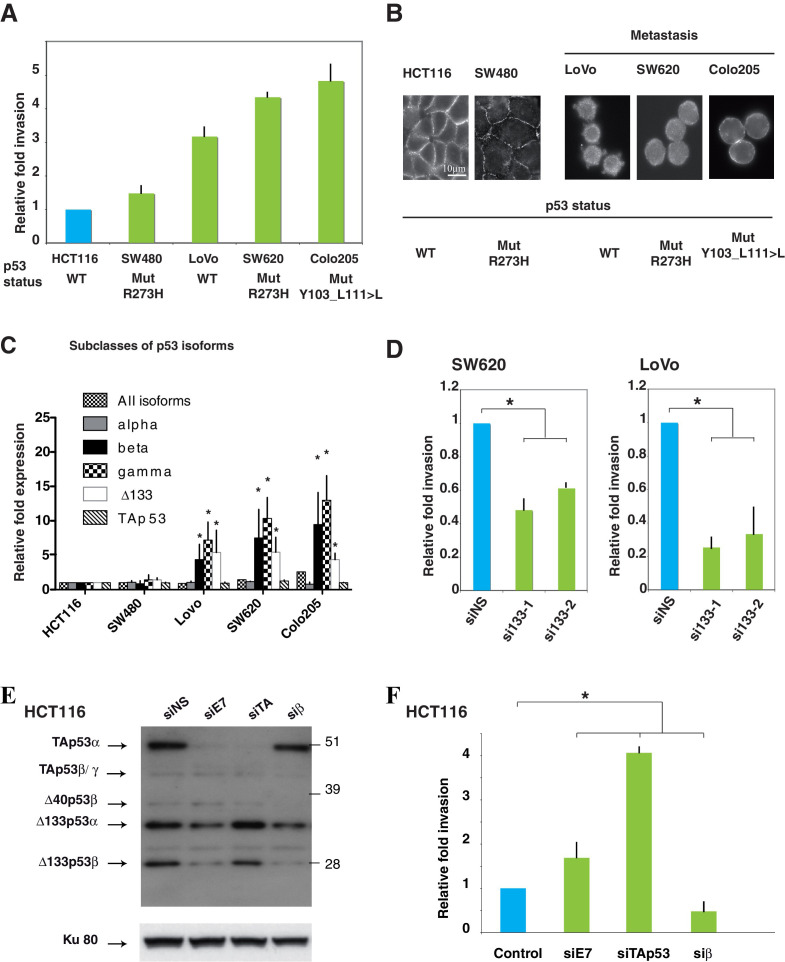


The article has been corrected accordingly.

